# Characteristics and Challenges of Poly(ethylene-*co*-vinyl acetate) Solution Electrospinning

**DOI:** 10.1021/acsomega.4c01452

**Published:** 2024-04-10

**Authors:** Laura Unverzagt, Oleksandr Dolynchuk, Olaf Lettau, Christian Wischke

**Affiliations:** † Institute of Pharmacy, Martin-Luther-University Halle-Wittenberg, Kurt-Mothes-Str. 3, Halle 06120, Germany; ‡ Institute of Physics, Martin-Luther-University Halle-Wittenberg, Halle 06120, Germany; § Institute of Functional Materials for Sustainability, Helmholtz-Zentrum Hereon, Teltow 14513, Germany

## Abstract

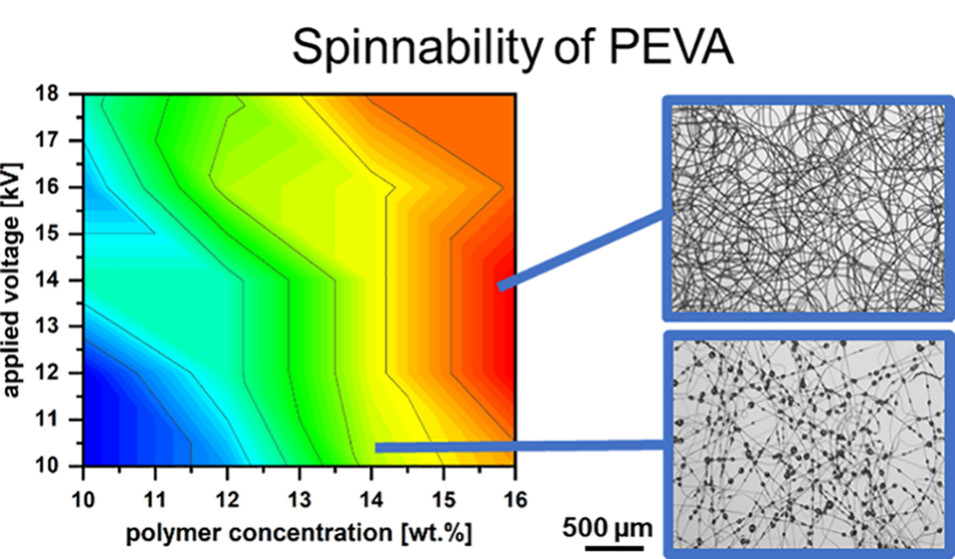

Poly(ethylene-*co*-vinyl acetate) (PEVA) is a versatile elastic, durable, and biocompatible copolymer, which can be processed by melt extrusion or solvent casting, while electrospinning has been reported as challenging. Here, a spinnability window should be identified using a total of 10 different PEVA materials with increasing vinyl acetate content (∼12–40 wt %) and molecular weights (∼60–130 kDa). Based on the solubility predictions by calculating Hansen solubility parameters, candidate solvents were experimentally evaluated. Spinning experiments with systematic alteration of solution composition and processing parameters revealed the causes of material deposition at the spraying nozzle and multijet spinning characteristics. By introducing a spinnability score that accounts for product characteristics and reproducibility, the spinnability of PEVA could be rationalized. Overall, it was demonstrated that PEVA solutions with an apparent viscosity of 920–3500 mPa·s can be spun to bead-free fibers of ∼10 μm. This size may allow suspension electrospinning of composite fibers in the future.

## 1 Introduction

Poly(ethylene-*co*-vinyl acetate) (PEVA) is a versatile thermoplastic copolymer with random ethylene and vinyl acetate (VA) units, which determine the material properties such as melting point and polarity. Based on comonomer contents, PEVA can be tailored for different processing techniques and applications given the effect on melting transition and deformation behavior.^1,2^ For instance, PEVA has been used in the plastic industry, e.g., for packaging and films,^3^ often considering it as a chloride-free alternative to poly(vinyl chloride). PEVA also has a high electrical resistivity as a bulk material, promoting its application as insulation of electrical cables in challenging technical settings, such as in nuclear plants.^4^ The ability of PEVA to be cross-linked to covalent polymer network structures, e.g., via gamma-irradiation^5^ or via chemical agents,^6^ has led to materials with further improved mechanical stability. Beyond that, PEVA networks have also been shown to exhibit shape-memory properties^7^ and a temperature-memory effect.^8^ The durability of PEVA, as well as its excellent suitability for thermoplastic melt-based processing including fiber spinning techniques^9^ or its suitability for impregnation of cotton textiles,^10^ has also opened up a market for PEVA fabrics for indoor and outdoor applications. Beyond the technical field, the versatile use of PEVA expands to life sciences, including the field of drug delivery systems. There are several PEVA-based pharmaceutical products in routine medical treatments (e.g., Implanon, contraceptive implant) as well as an increasing interest in the investigation of new drug-releasing implants.^11^

The processing of PEVA to the desired device shapes focuses primarily on melt extrusion or solvent casting techniques. These methodologies typically lead to products that are dense, i.e., have low porosity. However, particularly in the biomedical field, there is an interest in porous polymer structures such as nonwovens,^12^ which could be used as stand-alone devices^13^ or as flexible coatings^14^ of other types of implants to mediate cellular attachment, drug release, etc. Solution-based electrospinning is one of the most extensively explored polymer processing techniques to create nonwovens for experimental studies.^15^ At the same time, there are massive efforts to increase productivity and bring electrospinning to industrially relevant scales, e.g., via multijet setups based on nozzle arrays^16^ or umbellate spinnerets,^17^ or via nozzle-less spinning.^18^ The basis of these techniques is an electrohydrodynamic process, in which liquid droplets of a polymer solution are electrified and stretched, resulting in the ejection of charged jets of polymer solution. As the solvent evaporates from these jets, fibers of micro- to nanometer scale diameters are produced.^19^ The initiation and continuation of these jets determine if a spinning process can be realized and whether fiber meshes are formed.^20^

Relevant material properties and process parameters to reach electrospinning conditions and modulate fiber characteristics are not only the polymer concentration in solution, the used solvent and its evaporation rate, the viscosity of the solution, the surface tension, and the conductivity, but also instrumental parameters such as the applied voltage, flow rate, distance to collector, surrounding humidity, and temperature.^19,21^ The impact of process parameters has been investigated for various polymers, e.g. poly(L-lactide) (PLLA), poly(caprolactone) PCL, poly(vinyl alcohol) (PVA),^21^ and some models have been proposed to link material properties and processability.^22^ However, it is well-known in the field that suitable electrospinning parameters need to be individually identified for each respective polymer.

For PEVA, some studies in the 2000s reported its electrospinning for potential biomedical applications,^23−25^ but the data were limited and were not followed up over the last two decades. More recently, it was noted that for pure PEVA, in this case with a VA content of 28 wt %, electrospinning “is highly difficult in standard laboratory conditions”, which is why a blending with degradable polylactide was suggested.^26^ A key issue seemed to be the solubility of PEVA in solvents suitable for electrospinning, which requires further attention. Another study reported that PEVA fiber meshes can be produced by solution blow spinning, a spinning technique that utilizes a gas stream to create fibers such as via an airbrush gun.^27^ These examples highlight the interest in fiber production from PEVA solutions.

Therefore, in this study, a systematic approach should be applied eventually leading to the identification of process parameters characterizing the spinnability window of PEVA. For this purpose, 10 different PEVA materials of different compositions, molecular weights, and thermal properties should be investigated. Assisted by prediction and experimental determination of solubilities, a rationale for selecting process parameters based on solution characteristics should be established and challenges for continuous processing should be identified.

## 2 Materials and Methods

### 2.1 Materials

PEVA with 28 and 40 wt % of VA were obtained from PolyScience Inc., Warrington, USA (here referred to as PolyS28 and PolyS40). Elvax650Q (12 wt % VA), Elvax550 (15 wt % VA), Elvax460A (18 wt % VA), Elvax240A (28 wt % VA), Elvax260A (28 wt % VA), Elvax3182A (28 wt % VA), Elvax150 (32 wt % VA), and Elvax40W (40 wt % VA) were from Dow Chemical Company. All PEVAs were used as received. Chloroform ≥99.8% (≤50 ppm of H_2_O) and hexane (98%) were from Carl Roth (Karlsruhe, Germany). Chloroform D1 (with 0.03% tetramethyl silane standard) was from Sigma-Aldrich/Merck KGaA (Darmstadt, Germany), 1,1,1,3,3,3-hexafluoro-2-propanol (HFIP; 99%) was from Fluorochem Limited (Hadfield, UK), *n*-butyl acetate was from Dr. K. Hollborn & Söhne GmbH & Co. KG (Leipzig, Germany), and toluene was from ORG Laborchemie (Bunde, Germany).

### 2.2 Characterization of PEVA Properties

The determination of VA content of PEVA was performed by proton nuclear magnetic resonance (^1^H NMR) in deuterated chloroform (400 MHz Varian NMR spectrometer; Agilent Technologies). The molar VA content was calculated based on the NMR spectra as previously reported,^28^ using the CH signals of the VA repetitive unit at 4.90 ppm (1H) and the combined signals of CH_3_ (VA, 3H) and CH_2_ (VA, 2H; ethylene 4H) moieties at 0.39–2.49 ppm. The weight fraction of VA could subsequently be calculated based on the molecular weights of the ethylene and VA repetitive units.

For thermal analysis of PEVA, differential scanning calorimetry (DSC) measurements were performed with a power-compensated DSC 8000 from PerkinElmer equipped with the PerkinElmer Intracooler 2 for controlled cooling and heating. Samples were sealed in 20 μL aluminum pans and measured in a nitrogen gas atmosphere. Heat-flow rate data were obtained during heating and cooling the samples from −60 to 160 °C at a rate of 10 °C/min. The raw heat-flow rate data were corrected for the instrumental asymmetry and converted into the temperature dependencies of the apparent specific heat capacity *C*_*p*_ (*T*). The data were analyzed from the second heating run.

Gel permeation chromatography (GPC) analysis was carried out using the Agilent 1260 Infinity II GPC system (Polymer Standards Service GmbH, Mainz, Germany) equipped with a UV and a refractive index (RI) detector. Additionally, PSS SLD 700 MALS (Polymer Standards Service GmbH, Mainz, Germany) and PSS DVD1260 online viscometer (Polymer Standards Service GmbH, Mainz, Germany) detectors were employed to determine the absolute molar masses based on lighter scattering measurements and universal calibration (polystyrene standards: 580 g/mol and 975,000 g/mol; Polymer Standards Service GmbH, Mainz, Germany). The eluent, chloroform, was stabilized with ethanol (0.6–1%) and used at a 1 mL/min flow. 50 μL of sample solutions (4 mg in 1 mL chloroform containing 0.1% toluene as flow marker) was injected. Separation was accomplished using a Lux guard column, 10 μm, 50 mm × 8 mm ID and two SDV 10 μm, 300 mm × 8.0 mm ID analytical, linear XL columns (Polymer Standards Service GmbH, Mainz, Germany).

### 2.3 Model-Based and Experimental Evaluation of PEVA Solubility

In order to identify potentially good solvents for electrospinning of PEVA, the Hansen solubility parameters (HSPs) of PEVA were estimated based on its molecular structure by the “Yamamoto molecular breaking” group contribution method using the HSPiP software (version 5.4.05; C. Hansen, S. Abbott).

For semiquantitative experimental evaluation of solubility, the respective solvent such as chloroform was added at various quantities to PEVA (typically 100–600 mg) in a sealed glass vial. The samples were stirred at room temperature by a magnetic stirrer for at least 3 h, and the dissolution was visually monitored. For poorly soluble polymers (e.g., Elvax460A, Elvax550, and Elvax650Q), additional sonication was applied (Bandelin Sonorex ultrasonic bath) while controlling the temperature to stay below 50 °C.

### 2.4 Rheology Measurement

Polymer solutions were prepared and equilibrated as described above. The rheological behavior of all polymer solutions was measured with an oscillatory rheometer (MCR 302e, Anton Paar, Germany) with a cone–plate geometry (angle 1°) with a diameter of 50 mm at 20 °C. The shear rate was either kept at 10 s^–1^ (determination of apparent viscosity) or systematically altered between 0.1 and 1000 s^–1^ (shear thinning investigation). The measurement with constant shear rate of 10 s^–1^ were performed over 180 s, while recording data points every second. The mean value of the apparent viscosity was calculated afterward from all data points. To restrict the evaporation of chloroform from the sample, a hood (solvent trap) was used to cover the cone–plate setup. Chloroform was placed inside the trap to create a chloroform atmosphere.

### 2.5 Electrospinning of PEVA Solutions

After complete dissolution, all polymer solutions for electrospinning were set aside for equilibration at room temperature for 20 h until further use. The employed electrospinning setup (Spraybase, CAT000002, profector life science) was vertically oriented and consisted of a power supply (5–20 kV), a syringe pump, a positively charged nozzle holder, and a grounded collector plate (10 cm diameter). The distance from the nozzle (diameter 0.9 mm) to the collector was kept at 15 cm, and the collector was covered with aluminum foil. The PEVA solutions (concentration range of typically 4 to 16 wt % as detailed for the respective experiment in the Results and Discussion section) were pulled into 5 mL syringes, attached to the syringe pump, and maintained at ambient temperature while operating with a flow rate of 2 mL/h. The applied voltage was systematically altered in the range of 10–18 kV as indicated in the Results and Discussion section. Material built-up at the tip of the needle was removed with a tissue. Product samples were collected on thin glass sheets (18 mm × 18 mm × 0.15 mm), which were placed on the collector. Temperature and relative humidity were monitored and stayed between 20.5–28.3 °C and 34–56% RH, respectively, throughout the course of this study.

### 2.6 Characterization of Fiber Morphology

Electrospun PEVA samples were analyzed on thin glass sheets, which were quickly collected once a thin layer of material was deposited. The glass sheets were examined without further preparation by light microscopy (DMI6000B, Leica, Wetzlar, Germany), and fiber diameters were measured manually with ImageJ (version 1.53t). Additionally, for investigation via scanning electron microscopy (SEM), fiber meshes have been collected on aluminum foil and subjected to drying in a vacuum oven at room temperature for 24 h and afterward stored in a desiccator. Subsequent sample preparation for SEM included the cutting of 1 cm × 1 cm pieces out of the fiber mesh with the aluminum foil underneath and the attachment to the SEM sample holder with conductive adhesive stubs (Plano, Wetzlar, Germany). SEM images were taken without sputtering using a Phenom 2G Pro (Thermo Fisher Scientific, Darmstadt, Germany) with an applied voltage of 5 kV.

## 3 Results and Discussion

### 3.1 Characterization of PEVA Polymers

In this systematic study, a comprehensive set of PEVA materials should be evaluated for electrospinning, including polymers with a VA content of 12 up to 40 wt % (Table 1). Beyond composition, also the weight-average molecular weight (*M*_w_) was systematically altered, e.g., increasing from 54 to 129 kDa for PEVA with 28 wt % VA. The analysis of their thermal properties illustrated that all PEVAs had very broad melting transitions, with *T*_m_ decreasing with increasing VA content. The peak of the melting transition (*T*_m_) was above room temperature in all cases (Table 1). The glass transitions were below room temperature and were partially overlaid in DSC thermograms by the onset of melting. The investigation of thermal properties suggested that all the selected materials might be producing fibers that are elastic at ambient conditions.

**Table 1:** Structural and Morphological Characteristics of PEVA Materials Used in This Study

	Elvax650Q	Elvax550	Elvax460A	Elvax240A	Elvax260A	Elvax3182A	PolyS28	Elvax150	Elvax40W	PolyS40
VA_nominal_ [wt %]^a^	12	15	18	28	28	28	28	32	40	40
VA_NMR_ [wt %]^b^	12.6	14.3	18.1	27.9	27.9	27.3	27.4	31.7	39.0	37.6
*T*_g_ [°C]^c^	–32	–32	–29	f	f	f	f	f	f	f
*T*_m_ [°C]^c^	92	88	86	67	70	71	60	61	48	50
*M*_w_ [kDa]^d^	e	e	101	54	70	79	129	71	59	75
PD^d^	e	e	3.8	2.9	2.9	2.9	2.6	3.3	2.8	2.4

a According to product labels.

b Molar composition determined by ^1^H NMR and transformed to wt %.

c Determined by DSC.

d Determined by GPC in chloroform.

e Not analyzed due to insufficient solubility.

f Not analyzed due to overlay with melting transition.

### 3.2 Evaluation of PEVA Solubility

For successful solution electrospinning, the polymer of interest has to be sufficiently solvated, which has been mentioned to be a critical issue for PEVA.^26^ Therefore, in order to rationally identify the suitable solvents for PEVA, first, a model-based approach was chosen based on HSP. The HSP concept considers dispersion forces [δD], polar forces [δP], and hydrogen bond forces [δH] (always listed in that order [δD, δP, δH]). These HSPs should be utilized to predict polymer/solvent solubility and select solvents for subsequent experimental studies.

**Figure 1:**
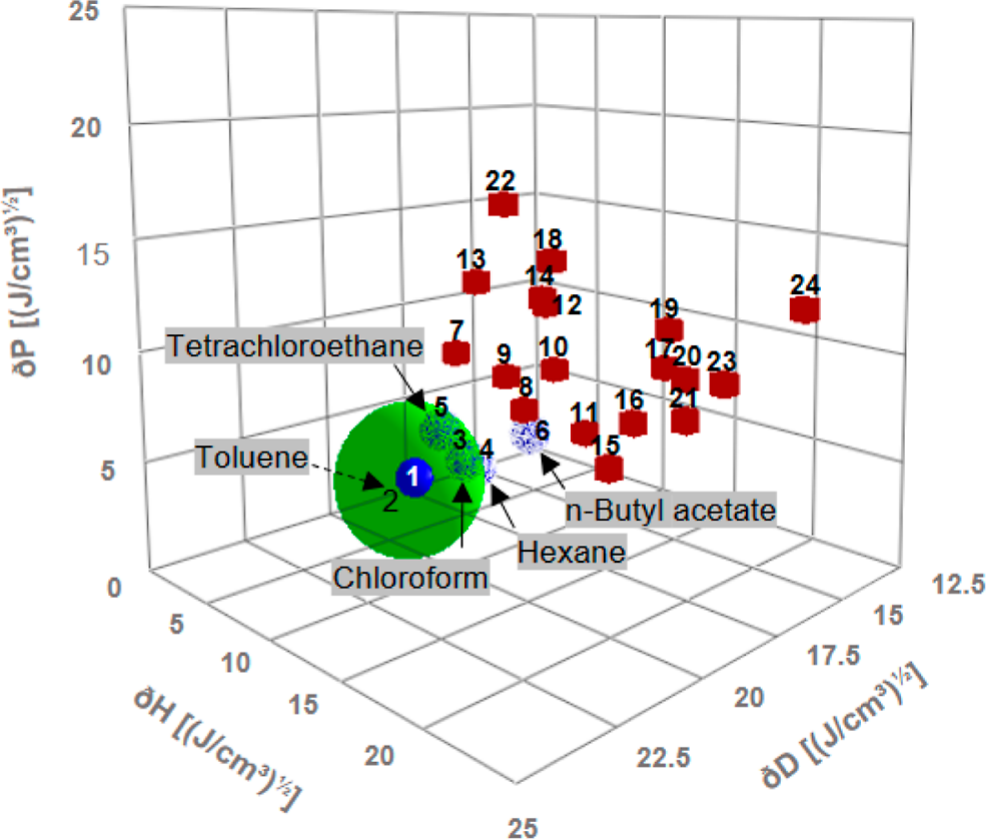
Hansen plot with estimated Hansen sphere as calculated by HSPiP software. Exemplary calculated data for PEVA with 28 wt % VA (1) and the following solvent candidates: (2) toluene, (3) chloroform, (4) hexane, (5) 1,1,2,2-tetrachloroethane, (6) *n*-butyl acetate, (7) cyclohexanone, (8) tetrahydrofuran, (9) methylene dichloride, (10) methyl acetate, (11) ethylene glycol monoethyl ether acetate, (12) acetone, (13) *n*-methyl-2-pyrrolidone, (14) *N*,*N*-dimethylacetamide, (15) hexafluoro isopropanol, (16) 1-pentanol, (17) acetic acid, (18) dimethylformamide, (19) formic acid, (20) 2,2,2-trifluoroethanol, (21) 1-propanol, (22) dimethyl sulfoxide, (23) ethanol, (24) methanol.

Based on the general chemical structure of PEVA and various relevant VA contents (12–40 wt %), the calculated HSPs were identified to slightly differ for the different PEVA compositions, particularly showing increasing contributions of polar forces and hydrogen bonding with increasing VA (Table 2). These computationally determined HSP estimates were graphically visualized in the Hansen plot (Figure 1) in order to illustrate their proximity to the HSP’s of potential solvents. In this study, 23 potential solvents were considered. All of these solvents have previously been used for electrospinning according to the literature.^26,29−35^ The potential suitability of a solvent to dissolve PEVA can be estimated by the distance of their HSP in the Hansen space, where good solvents should theoretically be located within the volume of the Hansen sphere of PEVA.

**Table 2:** Evaluation of PEVA Solubility Based on Predictions by HSP and Experimental Verification in Semi-Quantitative Solubility Tests for PolyS28

	Hansen solubility parameter [δD, δP, δH]	vapor pressure 25 °C [kPa]	experimental solubility of PolyS28 *c*_max_ [wt %]	comment
PEVA 12 wt % VA	[17.7, 0.6, 0.5]	n.a.^a^	n.a.^a^	-
PEVA 28 wt % VA	[17.8, 1.0, 1.1]	n.a.^a^	n.a.^a^	-
PEVA 40 wt % VA	[18.0, 1.4, 1.7]	n.a.^a^	n.a.^a^	-
toluene	[18.0, 1.4, 2.0]	3.8	22	highly viscous solution
chloroform	[17.8, 3.1, 5.7]	26	18	highly viscous solution
hexane	[14.9, 0.0, 0.0]	20	<1	slightly turbid at 1 wt %
1,1,2,2-tetrachloroethane	[18.8, 5.1, 5.3]	0.8	8	highly viscous solution
*n*-butyl acetate	[15.8, 3.7, 6.3]	1.6	2	low viscous solution
hexafluoroisopropanol	[17.2, 4.5, 14.7]	21	<1	insoluble

a Not applicable.

As the HSPs of PEVA were obtained by computational prediction only and thus also the radius of the PEVA sphere is set by default, here also some solvents with a fairly close distance [as stated in (J/cm^3^)^1/2^] to the PEVA target value [for 28 wt % VA] should be further considered even if not located within the PEVA sphere. The following top 5 solvents were chosen for further investigation: toluene [1.06 (J/cm^3^)^1/2^], chloroform [5.06 (J/cm^3^)^1/2^], hexane [5.99 (J/cm^3^)^1/2^], tetrachloroethane [6.20 (J/cm^3^)^1/2^], and *n*-butyl acetate [7.09 (J/cm^3^)^1/2^]. These data suggest that toluene might be the best solvent for PEVA with 28% VA. Given the common opinion that HFIP is a problem-fixing solvent for electrospinning, we also included HFIP (see Table 2) despite its larger distance of 14.09 (J/cm^3^)^1/2^ from PEVA in the Hansen space. While solution electrospinning requires a certain solubility of the polymer, it can be advantageous to use a moderately good rather than very good solvent to enforce polymer chain interaction during the spinning process.^36^

In the next step, the different solvent candidates have been evaluated experimentally for their ability to dissolve PEVA. In semiquantitative tests, the solvent was added to PEVA until no undissolved material was visually detectable. As shown in Table 2, the results are mostly in good agreement with the prediction of good dissolving power according to the HSPs. One exception was hexane, which did not dissolve PEVA properly due to the lack of polar forces and hydrogen bonding.

In addition to adequate solubility, vapor pressure is similarly important to ensure the evaporation of the solvent during the spinning processes and to create bead-free fibers. Hexane and chloroform are highly volatile compared to tetrachloroethane and toluene. Although the solubility of PEVA in chloroform is seemingly lower than in toluene, chloroform might be a strong candidate solvent due to the higher evaporation rate. As will be illustrated below, pure toluene was practically unsuitable for electrospinning of PEVA.

### 3.3 Characterization of PEVA Solutions

**Figure 2:**
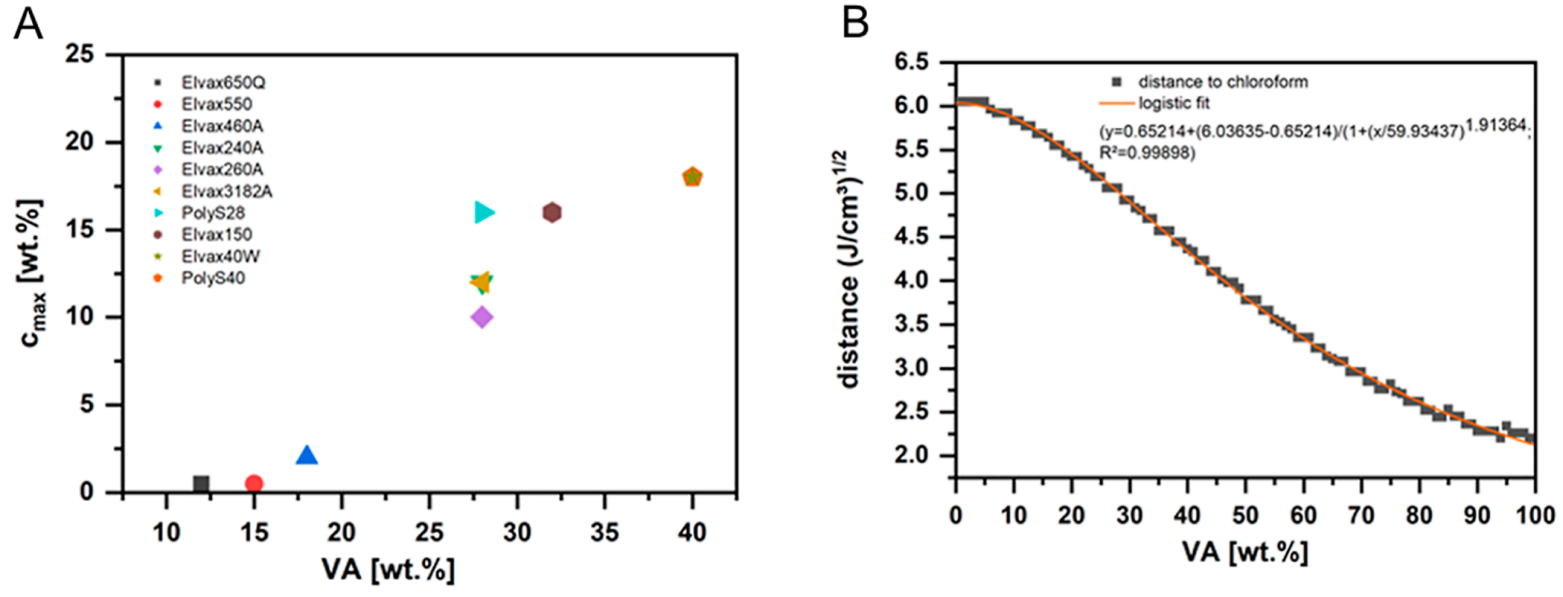
Properties of PEVA solutions depending on polymer composition. (A) Semiquantitative assessment of solubility in chloroform (maximum concentration that can be practically handled before gelling, *c*_max_) for different PEVA materials. (B) Calculated distance between the HSP of chloroform and PEVA with 0–100 wt % VA fitted as a logistic function.

Since polymer entanglement in a spinning solution is essential to go from a dripping regime to actual fiber production, relevant parameters accounting for such entanglements should be considered. First, the molecular weight contributes to polymer entanglement, where typically polymers with high molecular weight (>60 kDa) are used to produce bead-free fibers, unless polymer–polymer interaction can be modulated otherwise, e.g., by electrostatic forces or hydrogen bonds.^35^ As shown in Table 1, this molecular size range has been reached by the polymers employed here. Second, a solvent must be used providing sufficiently strong interactions with the polymer to allow for unfolding of individual chains from a coiled to an expanded state. The investigation on HSP (see Figure 1 and Table 2) helped to identify such solvents. Third, the polymer concentration must be sufficiently high; in consequence, more entanglements can be expected during acceleration of the jet in the electric field. Therefore, maximum solubilities of PEVA with increasing VA content were determined semiquantitatively in chloroform. The observed increasing solubility of PEVA in chloroform with increasing VA content (Figure 2A) can be justified by the higher polarity of VA and capacity for stronger hydrogen bonding. It is not surprising that materials of similar VA content may show slight differences in solubility in this analysis, considering potential effects of molecular weights and tacticity as well as the accuracy of the semiquantitative methodology. However, it is obvious that the experimental solubility data follow the general trend as suggested by the distance of the HSP of the respective PEVA composition from the HSP of chloroform (Figure 2B). Higher VA contents were associated with a higher proximity of polymer and solvent in the Hansen space and thus a higher solubility.

**Figure 3:**
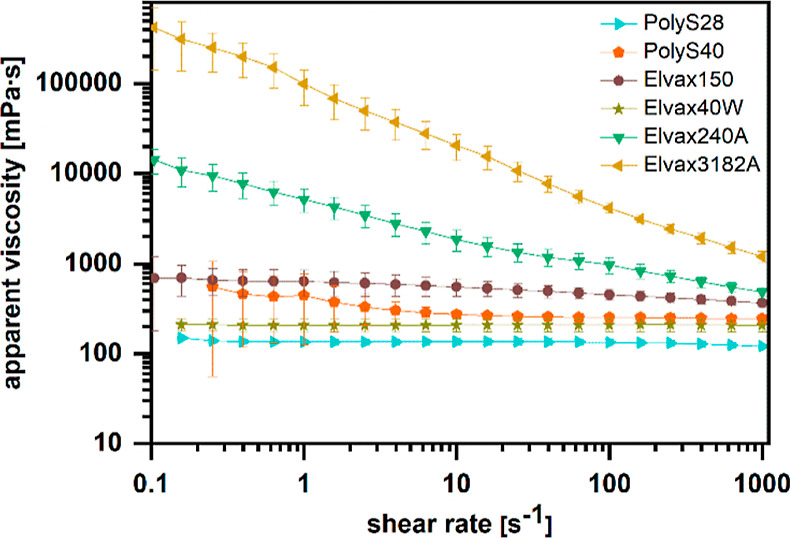
Shear thinning of 10 wt % PEVA solutions in chloroform at increasing shear rate as investigated by rotational rheology (mean of *n* = 3, error bars represent standard deviation).

Considering that electrospinning involves fluid transportation, the behavior under shear stress is an important factor for fiber production. Although the shear stress during feeding to the needle is relatively low (depending on tubing diameter etc.), the shear rate at the fluid cone drastically increases to a range of 100–1000 s^–1^,^37^ since static repulsion leads to jet formation. As investigated by rheology in shear rate sweep experiments for 10 wt % PEVA solutions in chloroform (PEVA materials with a solubility <10 wt % were not included in this experiment), some of the polymer solutions showed shear thinning with increasing shear rate, particularly Elvax240A and Elvax3182A (Figure 3). It is interesting to see that these polymers have identical VA content (27.4–27.9 wt %) but a lower *M*_w_ (54 and 79 kDa, respectively) than PolyS28 (129 kDa), which showed no shear thinning. At the same time, other materials in the lower molecular weight range but with higher VA content also showed no thinning in the studied shear rate range (Elvax150: 32 wt % VA; 71 kDa; Elvax40W: 39 wt % VA; 59 kDa). For PolyS40 (38 wt % VA; 77 kDa), a minor shear rate dependency of the apparent viscosity was observed (Figure 3). This indicates that beside molecular weight, further parameters can affect solution properties under shear, e.g., the ability for polymer–polymer interaction. Choosing a polymer solution that does not show shear thinning under the conditions of electrospinning can be crucial to obtain a reproducible fiber production, which has also been reported for polysaccharides earlier.^38^ In this respect, Elvax240A and Elvax3182A may require a further critical assessment.

### 3.4 Impact of Process Parameters on PEVA Solution Electrospinning

**Figure 4:**
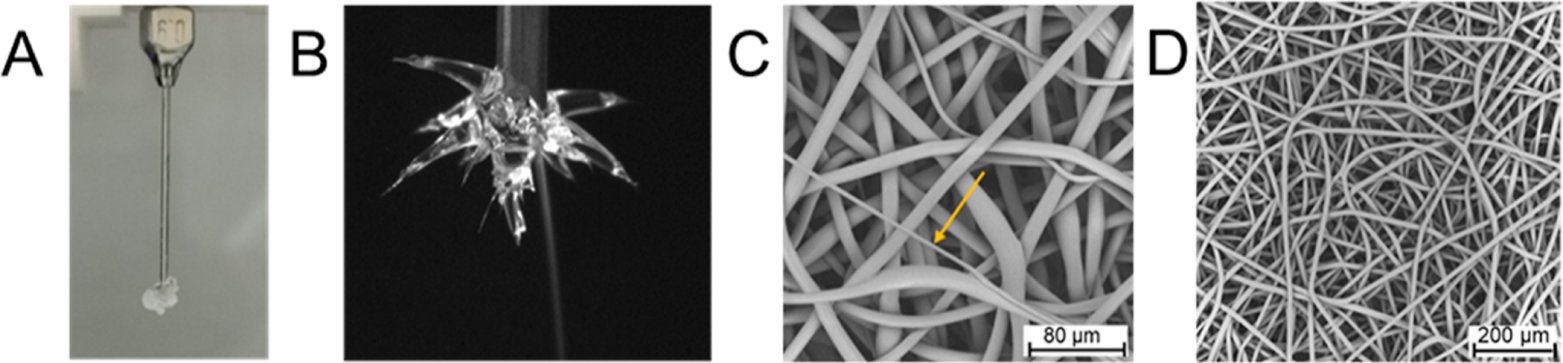
Challenges of PEVA electrospinning. (A) Exemplary image of nozzle with massive polymer deposition at the tip. (B) Characteristic image of PEVA spinning with dendritic multijet pattern originating from semisolid deposits of PEVA. (C) SEM image of PEVA fiber mesh, here showing conditions with a wide distribution of fiber diameters (PolyS40, 16 wt % in chloroform solution). (D) SEM image of PEVA fiber mesh with narrow distribution of fiber diameter (Elvax40W, 16 wt % in chloroform solution).

Electrospinning process parameters can be summarized into three main categories: (i) solution properties (polymer concentration/molecular weight, viscosity, solvent evaporation, etc.), (ii) experimental setup (voltage, flow rate, collector type/distance), and (iii) environmental conditions (humidity, temperature).^20^ It is an interplay of the above-mentioned parameters that ultimately allows the formation of fibers from the charged protruding meniscus of fluid at the tip of the feeding nozzle. This meniscus is typically shaped as a axisymmetric cone with a semivertical angle of 49.3° (Taylor cone),^39^ while also smaller semivertical angles^40^ as well as less symmetric cones have been reported.^41^ When spinning solutions of PEVA in chloroform, it was observed that the origin of fiber formation deviated from a cone-shaped meniscus. During the spinning process, the product was rapidly building up at the tip of the capillary, resulting in a spindle-like structure of semidried and dried polymer at the nozzle (Figure 4A). In order to explore the causes of this behavior, various tests were performed. First, electrospinning was performed on a different instrument (Fluidnatek LE-50, Bioinicia, Valencia, Spain) with a horizontal setup, resulting in comparable observations. Thus, an instrumental problem could be excluded. Second, the effect of solvent evaporation should be tested, which is faster with increasing solvent vapor pressure. In order to foster rapid evaporation, trials were performed with a coaxial needle setup (14 G within 20 G needle), where the outer port was flushed with compressed air (0.5–1 bar). Such additional airflow enhanced the evaporation of chloroform and thereby also amplified the product build-up at the nozzle. Similar observations were made when increasing the voltage or using higher polymer concentration. Overall, these experiments confirmed that the solvent evaporation from PEVA solutions in chloroform and the immediate PEVA precipitation [distance of HSP 5.06 (J/cm^3^)^1/2^] has been the major driving force for the observed product spindle. In consequence, third, strategies should be explored to reduce this phenomenon by reducing the solvent evaporation rates while still ensuring productivity of the process. Altering the feeding rates between 0.5 and 3 mL/h did not relevantly change the electrospinning characteristics of PEVA solutions, while a further reduction was not investigated for reasons of demanded process throughput. Given the very limited range of solvents suitable for PEVA (see Figure 1 and Table 2), the options for solvent exchange were strongly restricted to toluene. However, no sufficient electrospraying/-spinning process was observed for solutions of PEVA in toluene (15 wt % PolyS28 in toluene, 15 cm distance to collector, 0.9 mm nozzle, varied feeding rate 0.1–1 mL/h, varied voltage 10–18 kV). Instead, big droplets of polymer solution were observed on the collector, and no fibers could be formed. Alternatively, different amounts of toluene (10 and 25 wt %) were used to substitute chloroform in PEVA solutions. With 10 wt % toluene, smooth bead-free fibers could be formed, while at 25 wt % toluene, undesired beaded fibers appeared. The deposition of material at the nozzle was not relevantly affected when using chloroform/toluene mixtures compared to that with pure chloroform as the PEVA solvent. Overall, the addition of a low-volatile solvent was not significantly beneficial for the electrospinning process. Fourth, in order to complete the exploration of potential parameters, chloroform was substituted with polar fluids (enhanced conductivity) that could support the charging of the fluid meniscus and thus promote the transportation of PEVA from the nozzle toward the collector. Either dimethyl sulfoxide, dimethylformamide, or *N*,*N*-diisopropylethylamine (each 5 wt %) was added to PolyS28 in chloroform solution (total polymer concentration 14 wt %). Again, this parameter variation did not prevent the spindle-like material deposition at the nozzle. Based on these observations, it can be concluded that a lack of conductivity is not the reason for the buildup of product at the nozzle.

It should be emphasized that the electrospinning with the irregular fluid cone structure observed here did not hinder the fabrication of a fiber mesh. Instead, it led to dendritic jets or multiple jets eluting from the product at the tip of the nozzle (Figure 4B). When regularly removing any deposited polymer at the nozzle of vertical top–down electrospraying set-ups, a contamination of the formed fiber mesh by clots falling down from the nozzle can be prevented. Similar to electrospinning with a Taylor cone, surface tension must to be exceeded to initiate the jet formation. When semidried or dried polymer remains at the orifice, surface tension will locally vary, and therefore, jets with different diameter may appear, resulting in fibers with variable dimensions. This effect has been observed to various degrees with the different PEVA materials. For instance, Elvax240A or PolyS40 (Figure 4C) occasionally showed some fibers with an obviously smaller diameter, while no such very thin fibers were found in Elvax150 (14 wt %) or Elvax40W (16 wt %) samples (Figure 4D).

### 3.5 Optimal Spinnability Window of PEVA

**Figure 5:**
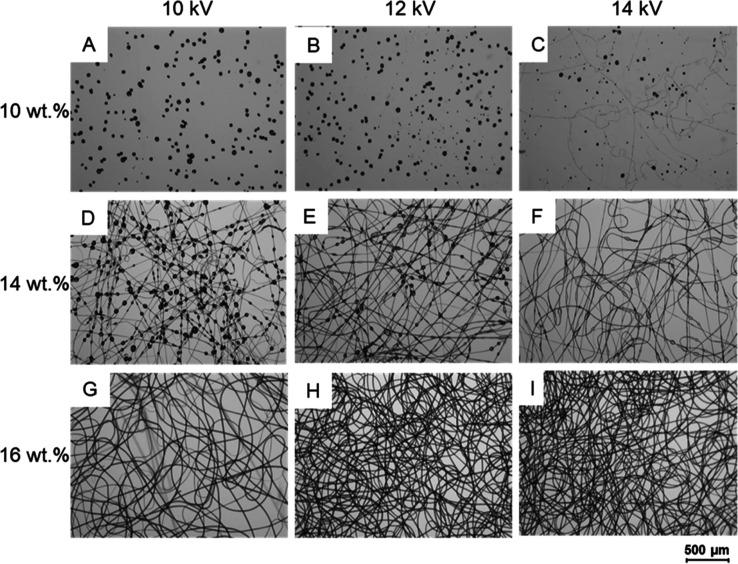
Impact of PEVA concentration and applied voltage on product characteristics. Light microscopy images of electrosprayed/-spun PolyS28 solutions in chloroform (A) 10 wt %, 10 kV: beads only, no fiber formation; (B) 10 wt %, 12 kV: beads with few isolated fibers; (C) 10 wt %, 14 kV: many beads with few fibers; (D) 14 wt %, 10 kV: strongly beaded fibers; (E) 14 wt %, 12 kV: beaded fibers; (F) 14 wt %, 14 kV: fibers with few beads; (G) 16 wt %, 10 kV: bead free fibers; (H) 16 wt %, 12 kV: bead-free fibers; (I) 16 wt %, 14 kV: bead-free fibers. All experiments: 15 cm distance to collector, 2 mL/h flow rate.

Considering all these previous results, an optimal spinnability window of chloroform solutions of PEVA should be identified in the next step. The effect of different combinations of polymer concentrations (10–16 wt %) and voltages (10–18 kV) on product characteristics was exemplarily shown for PolyS28 (28 wt % VA), while fixing the collector distance at 15 cm and the flow rate at 2 mL/h in these sets of experiments. By light microscopy of products collected on thin glass slides, it was obvious that increasing polymer concentrations and increasing voltage improved the fiber formation and reduced the number of beads contaminating the product (Figure 5).

**Figure 6:**
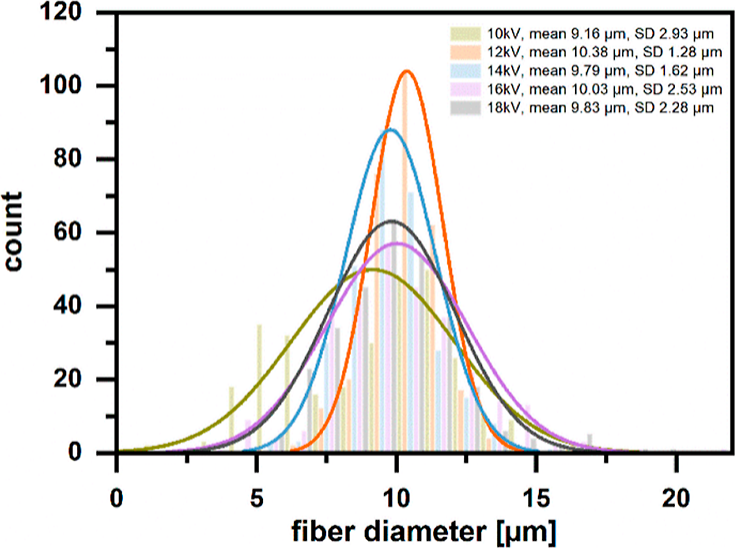
Size distribution of fiber diameters depending on the applied voltage during electrospinning of PolyS28 (16 wt % polymer solution in chloroform, 15 cm distance to collector, 2 mL/h flow rate). From microscopic images, 300 fiber diameters were measured with ImageJ for each processing condition. The data (colored columns) was fitted to a normal distribution with OriginPro for easier visualization (colored lines).

Considering our previous observation, that dendritic multijet electrospinning of PEVA from spindle-like cones can result in a certain width of the size distribution of fiber diameters, it was of interest to identify parameters leading to more homogeneous fiber dimensions. When light microscopy images of fibers (PolyS28) obtained from 16 wt % chloroform solutions were analyzed for fiber diameters, the narrowest distribution could be identified at 12 kV with a mean fiber diameter of 10.4 ± 1.28 μm (Figure 6).

**Figure 7:**
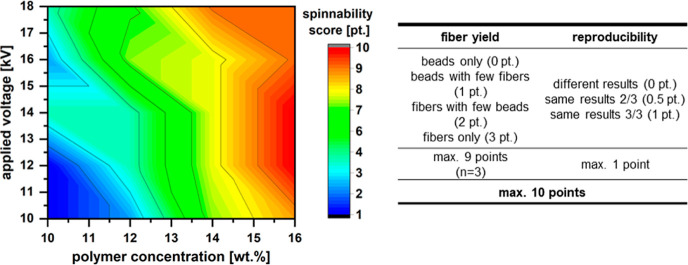
Spinnability score to assess fiber formation and reproducibility depending on polymer concentration and voltage. The data presented in a heat map are for PolyS28. The spinnability score was determined with 3 sets of repetitions of production (15 cm distance to collector, 2 mL/h flow rate) followed by an evaluation of the collected product according to the criteria shown in the table.

It is well accepted in the field of electrospinning, that for some materials, solvents, and ambient conditions, the day-to-day variability of spinning characteristics and thus the reproducibility of fiber products can be an issue. In order to include this aspect in the current study, a spinnability score (values 1–10; high scores desired) has been introduced that values both the bead-free fiber deposition and the day-to-day reproducibility (Figure 7). This spinnability score matrix was used to evaluate the optimal spinnability conditions for PolyS28, revealing the highest values for a polymer concentration of 16 wt % and medium voltage (12–14 kV) (Figure 7).

**Figure 8:**
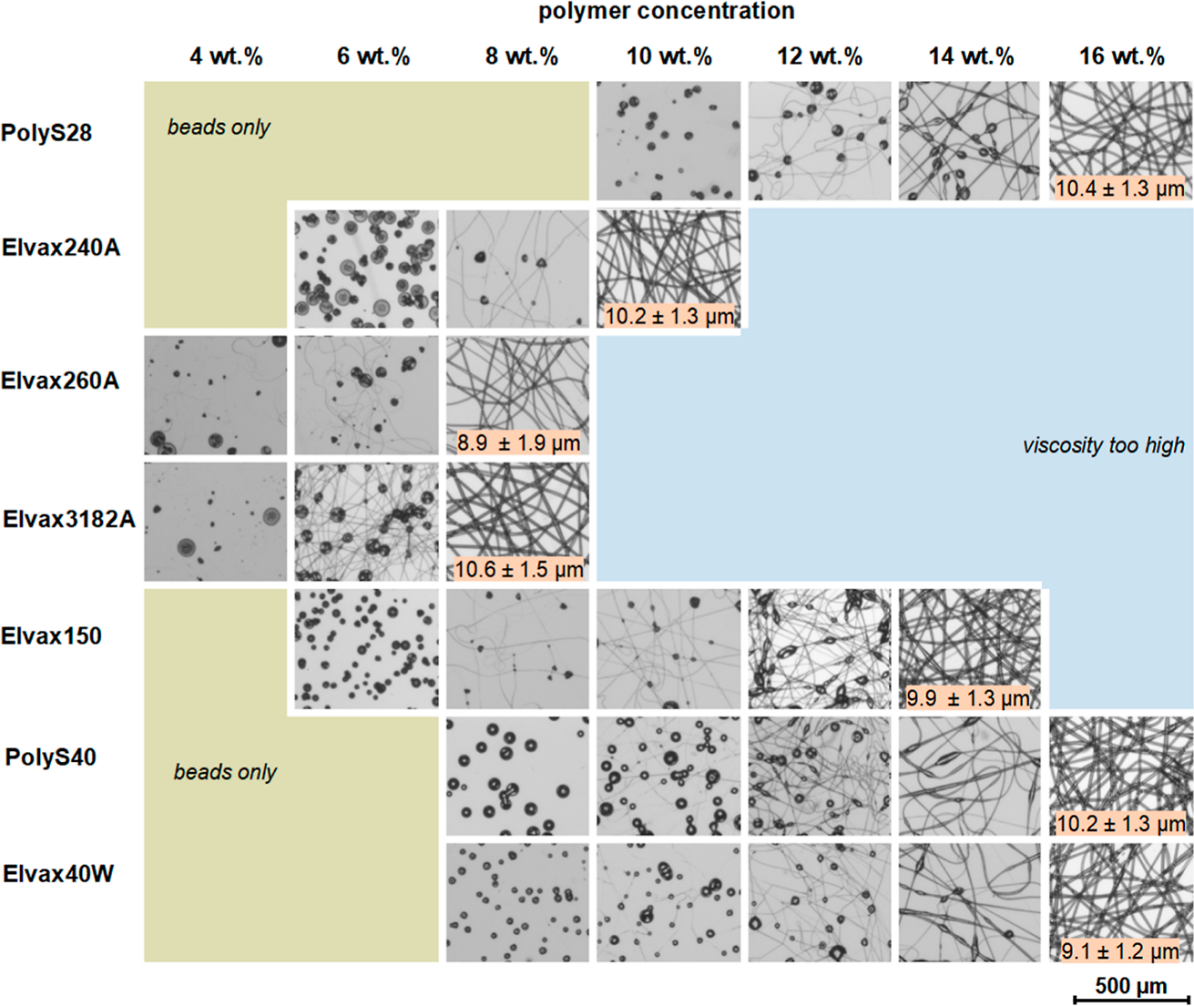
Spinnability window of various PEVA materials as illustrated by light microscopy images of collected products. Data presented for spinnable PEVA materials (PolyS28, Elvax240A, Elvax260A, Elvax3182A, Elvax150, PolyS40, and Elvax40W), while materials that did not result in fibers at any conditions are not shown (Elvax460A, Elvax550, and Elvax650Q). All materials were processed at 12 kV, 2 mL/h, 15 cm collector distance at varying concentrations in chloroform solution. Fiber diameters at the most suitable process condition are presented in the images [mean ± SD for *n* = 100 microscopically measured fibers (*n* = 300 for PolyS28)].

Subsequently, the parameter set of 12 kV, 2 mL/h, and collector distance of 15 cm was used to evaluate the spinnability of Elvax650Q, Elvax550, Elvax460A, Elvax240A, Elvax260A, Elvax3182A, Elvax150, Elvax40W, and PolyS40 at different polymer concentrations. It was not possible to spin Elvax650Q, Elvax550, and Elvax460A, all having low VA content and a low solubility in chloroform. Given this low polymer content in solution, only a spray deposition of particles and no fiber formation were observed. For Elvax240A, Elvax260A, Elvax3182A, Elvax150, Elvax40W, and PolyS40, the polymer concentration was varied in the range of 4–16 wt % depending on the respective solubility of the polymer (see Figure 2A). Like PolyS28, Elvax240A, Elvax260A, and Elvax3182A contain 28% VA but show different solubility in chloroform, forming highly viscous solutions at 16%, 10%, or 8 wt % respectively. Generally, an increase in polymer concentration promoted fiber formation, and it was possible to produce bead-free microscale fibers for all of those PEVAs (Figure 8). Regardless of which polymer was used, an average fiber diameter of 8.9–10.6 μm (Figure 8, see labels in figure) was microscopically determined for the optimum polymer concentrations resulting in bead-free nonwovens.

**Figure 9:**
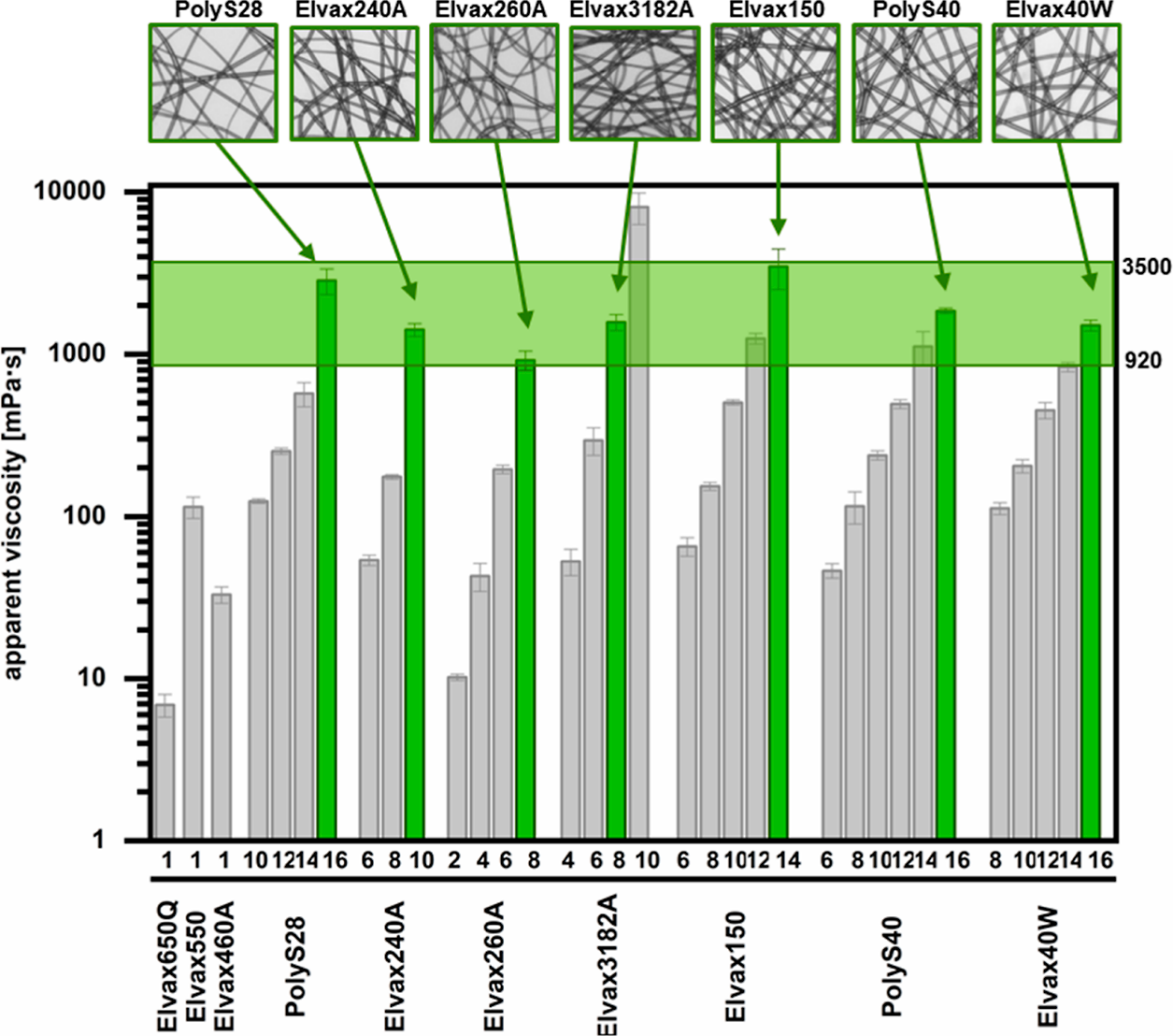
Apparent viscosity η_app_ of all PEVA solutions measured at 10 s^–1^. Labels at the *x*-axis indicate the respective type of PEVA and its concentration (1 to 16 wt %) in chloroform. The optimal polymer concentration for bead-free fibers is highlighted in green. Light microscopy images of fibers spun with optimal polymer concentration are shown in the top row. The green marked range of η_app_ includes all well electrospinnable PEVA solutions.

From the above presented data, it became obvious that neither the VA content of PEVA, nor the molecular weight, nor the polymer concentration was a good predictor for the spinnability of PEVA to bead-free fibers. For instance, Elvax240A (28% VA, 54 kDa) allowed producing excellent fibers from 10 wt % solutions, while PolyS28 (27% VA, 129 kDa) required 16 wt % solutions, which seems counterintuitive based on chain entanglement theories. Similar to PolyS28, also Elvax40W (39% VA, 59 kDa) and PolyS40 (38% VA, 77 kDa) allowed for bead-free fiber formation when using 16 wt % solutions in chloroform only, despite the VA content varying from PolyS28 and the molecular weight differing between these materials. Therefore, it would be relevant to identify a parameter (range) that is quantitative, easily accessible, and would be indicative of PEVA spinnability for various PEVA materials. In addition to shear thinning studies with shear rate sweeps (compare Figure 3), rheological experiments also allow collecting data for apparent viscosities η_app_ at constant shear rates (here: 10 s^–1^). When analyzing those rheological data for various PEVA materials and concentrations, a range of η_app_ spanning over 3 orders of magnitude from ∼10 to 10,000 mPa·s was determined. Importantly, when correlating η_app_ and successful fiber formation, it became obvious that a relatively narrow range of η_app_ of 920–3500 mPa·s was particularly suitable for the production of fibers for all PEVA materials irrespective of their VA content and molecular weights (Figure 9). This translates into different polymer concentrations in chloroform depending on the used polymer (optimal polymer concentration: PolyS28 16 wt %; Elvax240 10 wt %; Elvax260A and Elvax3182A 8 wt %; Elvax150 14 wt %; PolyS40 and Elvax40W at 16 wt %). Below this viscosity range, particles rather than fibers were formed, while above the range, streaked or torn fibers appeared. Therefore, for PEVA, the identified range of η_app_ might be an effective predictor of spinnability in solution electrospinning.

## 4 Conclusions

This study demonstrated that a variety of PEVA materials with a VA content of 28–40 wt % can be spun to create microscale fibers. Solution electrospinning of PEVA was characterized by a dendritic multijet cone, which increases the need for manual interference during the spinning process but does not hinder the formation of uniform fiber meshes. It was identified that an apparent viscosity range of 920–3500 mPa·s is a good predictor for spinnability, which similarly applied to various types of investigated PEVA materials. In the optimal parameter range, fiber meshes with a mean fiber diameter of 8.9–10.6 μm could be obtained from several PEVA types. Due to this moderate fiber diameter, PEVA is an optimal candidate for electrospinning of suspensions, allowing to create composite fibers in the future. Based on the presented spinnability score matrix and the knowledge of the spinnability window of PEVA, a specific PEVA material can be easily chosen in future studies to create interesting fiber-based materials.

## References

[ref1] Meszlenyi G.; Kortvelyessy G. Direct determination of vinyl acetate content of ethylene-vinyl acetate copolymers in thick films by infrared spectroscopy. Polym. Test. 1999, 18 (7), 551–557. 10.1016/S0142-9418(98)00053-1.

[ref2] Wang K.; Deng Q. The Thermal and Mechanical Properties of Poly(ethylene-co-vinyl acetate) Random Copolymers (PEVA) and its Covalently Crosslinked Analogues (cPEVA). Polymers 2019, 11 (6), 1055 10.3390/polym11061055. 31212957 PMC6631310

[ref3] Sonia A.; Priya Dasan K. Celluloses microfibers (CMF)/poly (ethylene-co-vinyl acetate) (EVA) composites for food packaging applications: A study based on barrier and biodegradation behavior. J. Food Eng. 2013, 118 (1), 78–89. 10.1016/j.jfoodeng.2013.03.020.

[ref4] Przybytniak G.; Boguski J.; Placek V.; Verardi L.; Fabiani D.; Linde E.; Gedde U. W. Inverse effect in simultaneous thermal and radiation aging of EVA insulation. eXPRESS Polym. Lett. 2015, 9 (4), 384–393. 10.3144/expresspolymlett.2015.36.

[ref5] Dalai S.; Wenxiu C. Radiation effects on poly(propylene) (PP)/ethylene-vinyl acetate copolymer (EVA) blends. J. Appl. Polym. Sci. 2002, 86 (13), 3420–3424. 10.1002/app.11365.

[ref6] Radhakrishnan C. K.; Sujith A.; Unnikrishnan G.; Thomas S. Effects of the blend ratio and crosslinking systems on the curing behavior, morphology, and mechanical properties of styrene–butadiene rubber/poly(ethylene-*co*-vinyl acetate) blends. J. Appl. Polym. Sci. 2004, 94 (2), 827–837. 10.1002/app.20939.

[ref7] Li F. K.; Zhu W.; Zhang X.; Zhao C.; Xu M. Shape memory effect of ethylene-vinyl acetate copolymers. J. Appl. Polym. Sci. 1999, 71 (7), 1063–1070. 10.1002/(SICI)1097-4628(19990214)71:7<1063::AID-APP4>3.0.CO;2-A.

[ref8] Kratz K.; Madbouly S. A.; Wagermaier W.; Lendlein A. Temperature-Memory Polymer Networks with Crystallizable Controlling Units. Adv. Mater. 2011, 23 (35), 4058–4062. 10.1002/adma.201102225. 21815223

[ref9] Yan Y.; Zhao Y.; Liu M. Influence of ethylene vinyl acetate on the spinnability and mechanical properties of poly(propylene)/zeolite-Ag blend fibers. J. Appl. Polym. Sci. 2005, 96 (4), 1460–1466. 10.1002/app.21589.

[ref10] Elbarbary A. M.; Elhady M. A.; Gad Y. H. Development of Cotton Fabrics via EVA/SiO2/Al2O3 Nanocomposite Prepared by γ-Irradiation for Waterproof and Fire Retardant Applications. J. Inorg. Organomet. Polym. Mater. 2022, 32 (10), 4039–4056. 10.1007/s10904-022-02395-w.

[ref11] Schneider C.; Langer R.; Loveday D.; Hair D. Applications of ethylene vinyl acetate copolymers (EVA) in drug delivery systems. J. Controlled Release 2017, 262, 284–295. 10.1016/j.jconrel.2017.08.004. 28789964

[ref12] Kim K.; Yu M.; Zong X.; Chiu J.; Fang D.; Seo Y. S.; Hsiao B. S.; Chu B.; Hadjiargyrou M. Control of degradation rate and hydrophilicity in electrospun non-woven poly(D,L-lactide) nanofiber scaffolds for biomedical applications. Biomaterials 2003, 24 (27), 4977–4985. 10.1016/S0142-9612(03)00407-1. 14559011

[ref13] Lopianiak I.; Wojasiński M.; Kuźmińska A.; Trzaskowska P.; Butruk-Raszeja B. A. The effect of surface morphology on endothelial and smooth muscle cells growth on blow-spun fibrous scaffolds. J. Biol. Eng. 2021, 15 (1), 27 10.1186/s13036-021-00278-1. 34924005 PMC8684665

[ref14] Wang J.; An Q.; Li D.; Wu T.; Chen W.; Sun B.; Ei-Hamshary H.; Al-Deyab S. S.; Zhu W.; Mo X. Heparin and Vascular Endothelial Growth Factor Loaded Poly(L-lactide-co-caprolactone) Nanofiber Covered Stent-Graft for Aneurysm Treatment. J. Biomed. Nanotechnol. 2015, 11 (11), 1947–1960. 10.1166/jbn.2015.2138. 26554154

[ref15] Ferrari G.; Thives Mello A.; Melo G.; de Mello Roesler C. R.; Salmoria G. V.; de Souza Pinto L. P.; de Mello Gindri I. Polymeric implants with drug-releasing capabilities: a mapping review of laboratory research. Drug Dev. Ind. Pharm. 2021, 47 (10), 1535–1545. 10.1080/03639045.2022.2043354. 35171071

[ref16] Ding B.; Kimura E.; Sato T.; Fujita S.; Shiratori S. Fabrication of blend biodegradable nanofibrous nonwoven mats via multi-jet electrospinning. Polymer 2004, 45 (6), 1895–1902. 10.1016/j.polymer.2004.01.026.

[ref17] Li H.; Chen H.; Zhong X.; Wu W.; Ding Y.; Yang W. Interjet distance in needleless melt differential electrospinning with umbellate nozzles. J. Appl. Polym. Sci. 2014, 131, 40515 10.1002/app.40515.

[ref18] Ebrahimi S.; Fathi M.; Kadivar M. Production and characterization of chitosan-gelatin nanofibers by nozzle-less electrospinning and their application to enhance edible film’s properties. Food Packag. Shelf Life 2019, 22, 100387 10.1016/j.fpsl.2019.100387.

[ref19] Xue J.; Wu T.; Dai Y.; Xia Y. Electrospinning and Electrospun Nanofibers: Methods, Materials, and Applications. Chem. Rev. 2019, 119 (8), 5298–5415. 10.1021/acs.chemrev.8b00593. 30916938 PMC6589095

[ref20] Rosic R.; Pelipenko J.; Kocbek P.; Baumgartner S.; Bešter-Rogač M.; Kristl J. The role of rheology of polymer solutions in predicting nanofiber formation by electrospinning. Eur. Polym. J. 2012, 48 (8), 1374–1384. 10.1016/j.eurpolymj.2012.05.001.

[ref21] El Fawal G. Polymer nanofibers electrospinning: A review. Egypt. J. Chem. 2020, 63 (4), 1279–1303. 10.21608/ejchem.2019.14837.1898.

[ref22] Shenoy S. L.; Bates W. D.; Frisch H. L.; Wnek G. E. Role of chain entanglements on fiber formation during electrospinning of polymer solutions: good solvent, non-specific polymer-polymer interaction limit. Polymer 2005, 46 (10), 3372–3384. 10.1016/j.polymer.2005.03.011.

[ref23] Kenawy E.-R.; Bowlin G. L.; Mansfield K.; Layman J.; Simpson D. G.; Sanders E. H.; Wnek G. E. Release of tetracycline hydrochloride from electrospun poly(ethylene-co-vinylacetate), poly(lactic acid), and a blend. J. Controlled Release 2002, 81 (1–2), 57–64. 10.1016/S0168-3659(02)00041-X. 11992678

[ref24] Sanders E. H.; Kloefkorn R.; Bowlin G. L.; Simpson D. G.; Wnek G. E. Two-Phase Electrospinning from a Single Electrified Jet: Microencapsulation of Aqueous Reservoirs in Poly(ethylene-co-vinyl acetate) Fibers. Macromolecules 2003, 36 (11), 3803–3805. 10.1021/ma021771l.

[ref25] Lewkowitz-Shpuntoff H. M.; Wen M. C.; Singh A.; Brenner N.; Gambino R.; Pernodet N.; Isseroff R.; Rafailovich M.; Sokolov J. The effect of organo clay and adsorbed FeO_3_ nanoparticles on cells cultured on Ethylene Vinyl Acetate substrates and fibers. Biomaterials 2009, 30 (1), 8–18. 10.1016/j.biomaterials.2008.09.015. 18838163

[ref26] Číková E.; Kuliček J.; Janigová I.; Omastová M. Electrospinning of Ethylene Vinyl Acetate/Poly(Lactic Acid) Blends on a Water Surface. Materials 2018, 11 (9), 1737 10.3390/ma11091737. 30223559 PMC6163409

[ref27] Rempel S. P.; Engler L. G.; Soares M. R. F.; Catafesta J.; Moura S.; Bianchi O. Nano/microfibers of EVA copolymer obtained by solution blow spinning: Processing, solution properties, and pheromone release application. J. Appl. Polym. Sci. 2019, 136 (24), 47647 10.1002/app.47647.

[ref28] Poljansek I.; Fabjan E.; Burja K.; Kukanja D. Emulsion copolymerization of vinyl acetate-ethylene in high pressure reactor-characterization by inline FTIR spectroscopy. Prog. Org. Coat. 2013, 76 (12), 1798–1804. 10.1016/j.porgcoat.2013.05.019.

[ref29] Luo C. J.; Nangrejo M.; Edirisinghe M. A novel method of selecting solvents for polymer electrospinning. Polymer 2010, 51 (7), 1654–1662. 10.1016/j.polymer.2010.01.031.

[ref30] Lu C.; Chen P.; Li J.; Zhang Y. Computer simulation of electrospinning. Part I. Effect of solvent in electrospinning. Polymer 2006, 47 (3), 915–921. 10.1016/j.polymer.2005.11.090.

[ref31] Lee K. H.; Kim H.; Khil M.; Ra Y.; Lee D. Characterization of nano-structured poly(ε-caprolactone) nonwoven mats via electrospinning. Polymer 2003, 44 (4), 1287–1294. 10.1016/S0032-3861(02)00820-0.

[ref32] Lasprilla-Botero J.; Alvarez-Lainez M.; Lagaron J. M. The influence of electrospinning parameters and solvent selection on the morphology and diameter of polyimide nanofibers. Mater. Today Commun. 2018, 14, 1–9. 10.1016/j.mtcomm.2017.12.003.

[ref33] Qian Y.-F.; Su Y.; Li X.-Q.; Wang H.-S.; He C. Electrospinning of Polymethyl Methacrylate Nanofibres in Different Solvents. Iran. Polym. J. 2010, 19 (2), 123–129.

[ref34] Denis P.; Dulnik J.; Sajkiewicz P. Electrospinning and Structure of Bicomponent Polycaprolactone/Gelatin Nanofibers Obtained Using Alternative Solvent System. Int. J. Polym. Mater. Polym. Biomater. 2015, 64 (7), 354–364. 10.1080/00914037.2014.945208.

[ref35] Wortmann M.; Frese N.; Sabantina L.; Petkau R.; Kinzel F.; Gölzhäuser A.; Moritzer E.; Hüsgen B.; Ehrmann A. New Polymers for Needleless Electrospinning from Low-Toxic Solvents. Nanomaterials 2019, 9 (1), 52 10.3390/nano9010052. 30609773 PMC6359487

[ref36] Ewaldz E.; Brettmann B. Molecular Interactions in Electrospinning: From Polymer Mixtures to Supramolecular Assemblies. ACS Appl. Polym. Mater. 2019, 1 (3), 298–308. 10.1021/acsapm.8b00073.

[ref37] Han T.; Yarin A. L.; Reneker D. H. Viscoelastic electrospun jets: Initial stresses and elongational rheometry. Polymer 2008, 49 (6), 1651–1658. 10.1016/j.polymer.2008.01.035.

[ref38] Stijnman A. C.; Bodnar I.; Hans Tromp R. Electrospinning of food-grade polysaccharides. Food Hydrocolloids 2011, 25 (5), 1393–1398. 10.1016/j.foodhyd.2011.01.005.

[ref39] Taylor G.I. Disintegration of water drops in an electric field. Proc. R. Soc. Lond. Ser. A: Math. Phys. Sci. 1964, 280 (1382), 383–397. 10.1098/rspa.1964.0151.

[ref40] Reneker D. H.; Yarin A.; Zussman E.; Xu H. Electrospinning of nanofibers from polymer solutions and melts. Adv. Appl. Mech. 2007, 41, 43–346. 10.1016/S0065-2156(07)41002-X.

[ref41] Abdulhussain R.; Adebisi A.; Conway B. R.; Asare-Addo K. Electrospun nanofibers: Exploring process parameters, polymer selection, and recent applications in pharmaceuticals and drug delivery. J. Drug Delivery Sci. Technol. 2023, 90, 105156 10.1016/j.jddst.2023.105156.

